# Improving the Reliability of Network Metrics in Structural Brain Networks by Integrating Different Network Weighting Strategies into a Single Graph

**DOI:** 10.3389/fnins.2017.00694

**Published:** 2017-12-19

**Authors:** Stavros I. Dimitriadis, Mark Drakesmith, Sonya Bells, Greg D. Parker, David E. Linden, Derek K. Jones

**Affiliations:** ^1^Division of Psychological Medicine and Clinical Neurosciences, School of Medicine, Cardiff University, Cardiff, United Kingdom; ^2^Cardiff University Brain Research Imaging Centre, School of Psychology, Cardiff University, Cardiff, United Kingdom; ^3^School of Psychology, Cardiff University, Cardiff, United Kingdom; ^4^Neuroinformatics Group, Cardiff University Brain Research Imaging Centre, School of Psychology, Cardiff University, Cardiff, United Kingdom; ^5^Neuroscience and Mental Health Research Institute, Cardiff University, Cardiff, United Kingdom; ^6^MRC Centre for Neuropsychiatric Genetics and Genomics, School of Medicine, Cardiff University, Cardiff, United Kingdom

**Keywords:** connectome, diffusion MRI, structural brain network, tractography, reliability

## Abstract

Structural brain networks estimated from diffusion MRI (dMRI) via tractography have been widely studied in healthy controls and patients with neurological and psychiatric diseases. However, few studies have addressed the reliability of derived network metrics both node-specific and network-wide. Different network weighting strategies (NWS) can be adopted to weight the strength of connection between two nodes yielding structural brain networks that are almost fully-weighted. Here, we scanned five healthy participants five times each, using a diffusion-weighted MRI protocol and computed edges between 90 regions of interest (ROI) from the Automated Anatomical Labeling (AAL) template. The edges were weighted according to nine different methods. We propose a linear combination of these nine NWS into a single graph using an appropriate diffusion distance metric. We refer to the resulting weighted graph as an Integrated Weighted Structural Brain Network (ISWBN). Additionally, we consider a topological filtering scheme that maximizes the information flow in the brain network under the constraint of the overall cost of the surviving connections. We compared each of the nine NWS and the ISWBN based on the improvement of: (a) intra-class correlation coefficient (ICC) of well-known network metrics, both node-wise and per network level; and (b) the recognition accuracy of each subject compared to the remainder of the cohort, as an attempt to access the uniqueness of the structural brain network for each subject, after first applying our proposed topological filtering scheme. Based on a threshold where the network level ICC should be >0.90, our findings revealed that six out of nine NWS lead to unreliable results at the network level, while all nine NWS were unreliable at the node level. In comparison, our proposed ISWBN performed as well as the best performing individual NWS at the network level, and the ICC was higher compared to all individual NWS at the node level. Importantly, both network and node-wise ICCs of network metrics derived from the topologically filtered ISBWN (ISWBN^TF^), were further improved compared to the non-filtered ISWBN. Finally, in the recognition accuracy tests, we assigned each single ISWBN^TF^ to the correct subject. We also applied our methodology to a second dataset of diffusion-weighted MRI in healthy controls and individuals with psychotic experience. Following a binary classification scheme, the classification performance based on ISWBN^TF^ outperformed the nine different weighting strategies and the ISWBN. Overall, these findings suggest that the proposed methodology results in improved characterization of genuine between-subject differences in connectivity leading to the possibility of network-based structural phenotyping.

## Introduction

Tractography is a popular method for extracting white matter connectivity from diffusion MRI (dMRI) and plays a key role in structural brain connectomics (Fornito et al., 2015). A variety of algorithms have been proposed, with the majority of them using voxel-based assessment of water diffusion to reveal paths/tracts of the white matter bundles. A fundamental problem with tractography is that there is no “ground truth” so it is impossible to separate “true” from spurious false positive and false negative connections (Smith et al., 2012; de Reus and van den Heuvel, 2013; Girard et al., 2014). Any noise in the system can lead to noisy connection matrices, particularly at the single-subject level, leading to numerous false positives (Thomas et al., 2014). It has recently been estimated that false positives are twice as detrimental as false negatives for any network metric derived from binary networks (Zalesky et al., 2016).

Two recent studies have attempted to solve this issue, which is a main obstacle for the application of graph theory to structural brain networks. Drakesmith et al. (2015b), proposed the multi-threshold permutation correction to overcome the effects of false positives and threshold bias. Roberts et al. proposed a consistent thresholding of structural brain networks that attempted to identify highly consistent and highly inconsistent subnetworks across subjects in a targeted cohort (Roberts et al., 2016).

One solution to this bias in structural brain connectivity metrics is to aggregate data over large samples of subjects as a way of increasing the signal to noise ratio, for example, through averaging of brain networks across subjects (Hagmann et al., 2008; Perry et al., 2015). An alternative to this group-averaging approach is to construct a consensus brain network by pooling edges that are derived from a predefined fraction of subjects across the whole cohort (van den Heuvel and Sporns, 2011; de Reus and van den Heuvel, 2013). Consensus brain network is a term derived from consensus clustering where different clusterings that have been obtained from the same dataset, after applying different clustering algorithms, are aggregated to fit a more robust/consistent clustering. Similarly, a consensus brain network maintains the edges that are highly representative across the cohort as a “majority vote” rule.

These aforementioned approaches are problematic because densely seeded tractography leads to dense structural brain networks and thus, a high level of inherent (but potentially spurious) overlap across subjects. The most common approach to tackling this issue is to adopt a “topological filtering” approach or a “threshold” in order to uncover the backbone of the network topology. Apart from reducing spurious connections, topological filtering of brain connectivity matrices plays a significant role in extracting connection topology (Bullmore and Bassett, 2011). The most common method in this setting is to “threshold” networks to some desired density by keeping only the “strongest” links (Dimitriadis et al., 2010). We recently proposed a data-driven topological filtering scheme based on orthogonal minimal spanning trees (OMST) (Dimitriadis et al., 2017b). It is extremely important that any data-driven filtering approach considers the topology of the brain network and treats both weak and strong connections equally (Gigandet et al., 2008).

Thresholding is widely used in both structural and functional brain network analysis as a step for binarizing the weighted networks (i.e., transforming them into unweighted networks (Dimitriadis et al., 2010, 2015a, 2016a,b,c,d; Rubinov and Sporns, 2010; Antonakakis et al., 2016). While such binarization procedures are recommended for separating strong from weak connections, they are not ideally suited to extracting network metrics. The relative weights on different edges are informative and can give a better characterization of the underlying structural and/or functional topology, potentially leading to better separation of groups or conditions.

Previous studies have attempted to reveal the reliability of network and node-wise network metrics for structural brain networks, using a few edge-weighting strategies. Cheng et al. (2012) assessed test-retest reliability using diffusion tensor MRI (DT-MRI) data from 44 subjects with a focus on the differences between binary and weighted networks. Buchanan et al. (2014), with repeated scans from nine subjects, explored the reliability of network metrics on a network and node-wise level using dMRI and two alternative tractography algorithms, two alternative seeding strategies, a white matter way point constraint and three alternative network weightings (Buchanan et al., 2014). Specifically, Cheng et al. (2012) explored variability of network metrics using DTI and two different weighting network strategies (WS). In the first approach, the weights were computed as the ratio between the sum of the inverse of the fiber length and the mean volume of two Regions of Interests (ROIs) (WS1), while in the second (WS2), they eliminated the fiber length, counting only the number of fibers normalized by the sum of the voxels in both ROIs. The Intra-Class Correlation Coefficients (ICCs) for six network metrics varies from 0.54 to 0.67 for WS1 but varies from 0.3 to 0.64 for WS2. Buchanan et al. (2014) reported global within-subject differences between 3.2 and 11.9%, with ICCs between 0.62 and 0.76. The mean nodal within-subject differences were between 5.2 and 24.2%, with mean ICCs between 0.46 and 0.62. For 83.3% (70/84) of nodes, the within-subject differences were smaller than between-subject differences.

Both studies demonstrated (ICCs) for network-wise network metrics using a few edge-weighting strategies. However, they did not assess the reliability of network metrics at a node level and more importantly, did not propose a solution for further improving the reliability of the existing methodology in constructing structural brain networks.

In this study, we constructed structural brain networks from five repeat scans of five healthy volunteers by adopting nine different network weighting strategies (NWS) affecting the construction of networks. In each of the nine alternative network weighting scenarios DT-MRI-based weights (Fractional Anisotropy-FA/Radial Diffusivity-RD/Mean Diffusivity-MD), average tract length (ATL), Euclidean distance between the coordinates of the ROIs (ED), the volume of the tract (TV), the number of streamlines (NSTR) and the proportion of streamlines (PSTR) [see section Network Weighting Strategies (NWS) for the definition], we quantified the reliability of six graph-theoretic measures network-wise (characteristic path length, global/local efficiency, radius, diameter, and eccentricity) and two node-wise (global and local efficiency) using (ICC). Since these measures are essential prerequisites for characterizing complex networks, their reliability is crucial to the ultimate interpretation of structural brain networks. Additionally, we propose a methodology for combining the alternative network weighted brain networks into a single integrated weighted structural brain network (IWSBN). We compared the ICCs of the same network metrics both network and node-wise, derived from the IWSBN, with those derived using the nine individual NWS. We also present a data-driven thresholding scheme that can extract the backbone of structural brain networks by optimizing the information flow under the constraint of the overall cost of the selected weighted connections. This topological filtering scheme was applied to the IWSBN, and the ICCs of the network metrics were again estimated. Finally, we tested the NWS-based weighted brain networks, the proposed IWSBN and its topologically filtered version IWSBN^TF^ in terms of the ability to match each network to the correct subject out of the whole cohort (i.e., identify which networks are derived from repeat scans of the same subject, which we refer to as “recognition accuracy”). This is important, as it captures the ability to separate intra-individual differences in derived networks (where variance derives from measurement noise), from interindividual differences in networks (reflecting true underlying biological differences). As such, this facilitates the study of individualized structural brain networks without having to resort to group-averaging approaches.

## Materials

### Participants

In total, five healthy subjects participated in this pilot study (mean 37.1 ± 4.9 years std age, five males). The whole procedure involved five repeat scans for each participant 1 week apart from each other. All participants were recruited through the School of Psychology, Cardiff, Wales, UK. All participants were undergoing or had previously completed a university degree course, were right handed as assessed with the Edinburgh Handedness Inventory^3^ and of Caucasian origin. Exclusion criteria included a current episode or a history of neurological and psychiatric disorders, drug or alcohol abuse and medication that may have an impact on the structure of the brain. For assessment, the general health questionnaire was used (Goldberg and Huxley, 1980). All subjects provided a written informed consent.

### Structural MRI scanning

T_1_-weighted structural scans were acquired using an oblique axial, 3D fast-spoiled gradient recalled sequence (FSPGR) with the following parameters: TR = 7.9 ms, TE = 3.0 ms, inversion time = 450 ms, flip angle = 20°, 1 mm isotropic resolution, with a total acquisition time of ~7 min.

### Diffusion MRI scanning

High angular resolution diffusion-weighted imaging (HARDI) data were acquired in the Cardiff University Brain Research Imaging Centre (CUBRIC) on a 3 T GE Signa HDx system (General Electric, Milwaukee, USA) using a cardiac-gated, peripherally gated twice-refocused spin-echo Echo Planar Imaging (EPI) sequence, with effective TR/TE of 15R-R intervals/87 ms. Sets of 60 contiguous 2.4 mm thick axial slices were obtained, with diffusion-sensitizing gradients applied along 30 isotropically distributed (Jones et al., 1999) gradient directions (*b* = 1,200 s/mm^2^). For further details of the MRI protocol see (Bracht et al., 2016).

### Diffusion MRI data preprocessing

Data were analyzed using Explore DTI 4.8.3 (Leemans et al., 2009). Eddy-current induced distortion and motion correction was performed using an affine registration to the non-diffusion-weighted B_0_-images, with appropriate re-orienting of the encoding vectors (Leemans and Jones, 2009). Field inhomogeneities were corrected for using the approach of Wu et al. (2008). The diffusion-weighted images (DWIs) were non-linearly warped to the T_1_-weighted image using the FA map, calculated from the DWIs, as a reference. Warps were computed using Elastix (Klein et al., 2010) normalized mutual information as the cost function and constraining deformations to the phase-encoding direction. The corrected DWIs were therefore transformed to the same (undistorted) space as the T_1_-weighted structural images. A single diffusion tensor model was fitted to the diffusion data in order to compute quantitative parameters such as FA (Basser et al., 1994). Following the method of Pasternak et al. (2009), a correction for free water contamination of the diffusion tensor based estimates was applied (Pasternak et al., 2009; Metzler-Baddeley et al., 2012). Data quality was checked by careful visual inspection and by looking at the average residuals per DWI for each participant.

### Tractography

DT-MRI analysis was performed using ExploreDTI (Leemans et al., 2009) following peaks in the fiber orientation density function (fODF) reconstructed from the damped Richardson Lucy algorithm (dRL) (Dell'acqua et al., 2010; Jeurissen et al., 2013). The dRL algorithm estimates multiple fiber orientations in a single voxel and therefore provides a more accurate diffusion profile than DT-MRI-based methods estimating only one fiber orientation per voxel. For each voxel in the dataset, streamlines were initiated along any peak in the (fODF) that exceeded an amplitude of 0.05. A streamline, uniform step-size, algorithm based on that of Basser et al. (2000), but extended to multiple fiber orientations within each voxel (Jeurissen et al., 2011), was used for tractography. Each streamline continued in 0.5 mm steps following the peak in the fODF that subtended the smallest angle to the incoming trajectory. Termination criteria were an angle threshold >45° and fODF amplitude <0.05.

### Network construction

The automated atlas labeling (AAL) atlas (Tzourio-Mazoyer et al., 2002) was registered to the HARDI data using a nonlinear transformation (Klein et al., 2010). The streamline termination points were coregistered to each AAL region. The numbers of streamlines connecting each pair of AAL regions were aggregated into a 90 × 90 connectivity matrix.

Connections between regions were computed by identifying the streamlines connecting each pair of gray matter ROIs. The endpoint of a streamline was considered to be the first gray matter ROI encountered when tracking from the seed location

Streamlines that did not connect to an ROI were discarded. Networks were computed for 13 different thresholds of streamline filtering by minimum contiguous length in white matter, from 0 to 6.0 mm in increments of 0.5 mm (Buchanan et al., 2014). For instance, a threshold of l mm discards any streamline that does not pass through at least l mm in white matter between gray matter ROIs.

### Network weighting strategies (NWS)

In this section, we describe the nine adopted NWS derived from tractography.

Fractional anisotropy (FA) is calculated from the eigenvalues (λ_1_, λ_2_, λ_3_) of the diffusion tensor. The eigenvectors (ϵ) give the orientations in which the ellipsoid has major axes and the corresponding eigenvalues give the magnitude of the peak along each axis (Basser and Pierpaoli, 1996). The mean diffusivity (MD) is the average of the three eigenvalues, while the axial and radial diffusivity are given by the largest and average of the two smallest eigenvalues, respectively (Basser et al., 1994).

The fourth NWS was based on average streamline tract length (ATL) leading to ATL-weighted networks. The fifth NWS estimated the Euclidean distance (ED) between the centroids of the two ROIs leading to the ED-weighed network. The Euclidean distance is computed in native space, so will vary across individuals.

The sixth NWS, termed streamline density (SD-weighted), records the interconnecting streamline density corrected for ROI size:

(1) $$w_{ij}=\frac{2}{g_{i}+g_{j}}\left|S_{ij}\right|$$

where *S*_*ij*_ is the set of all streamlines found between node *i* and node *j* (and *S*_*ij*_ = *S*_*ji*_), and *g*_*i*_ and *g*_*j*_ are the number of gray matter voxels in nodes *i* and *j*. This approach leads to the construction of a SD-weighed network.

The seventh NWS is based on the volume of the tract (TV) leading to TV-weighted networks. The tract volume is computed by counting the number of voxels the streamlines of a bundle occupy and multiplying by the voxel size.

Two further NWSs were based on the number and the percentage of streamlines that connected a pair of ROIs. The number of streamlines (NSTR) is the absolute NSTR connecting two regions. The proportion of streamlines (PSTR) is the NSTR between each pair of regions, normalized to the total NSTR across the whole brain.

The adopted NWSs are called the NSTR and PSTR.

**Figure 1:**
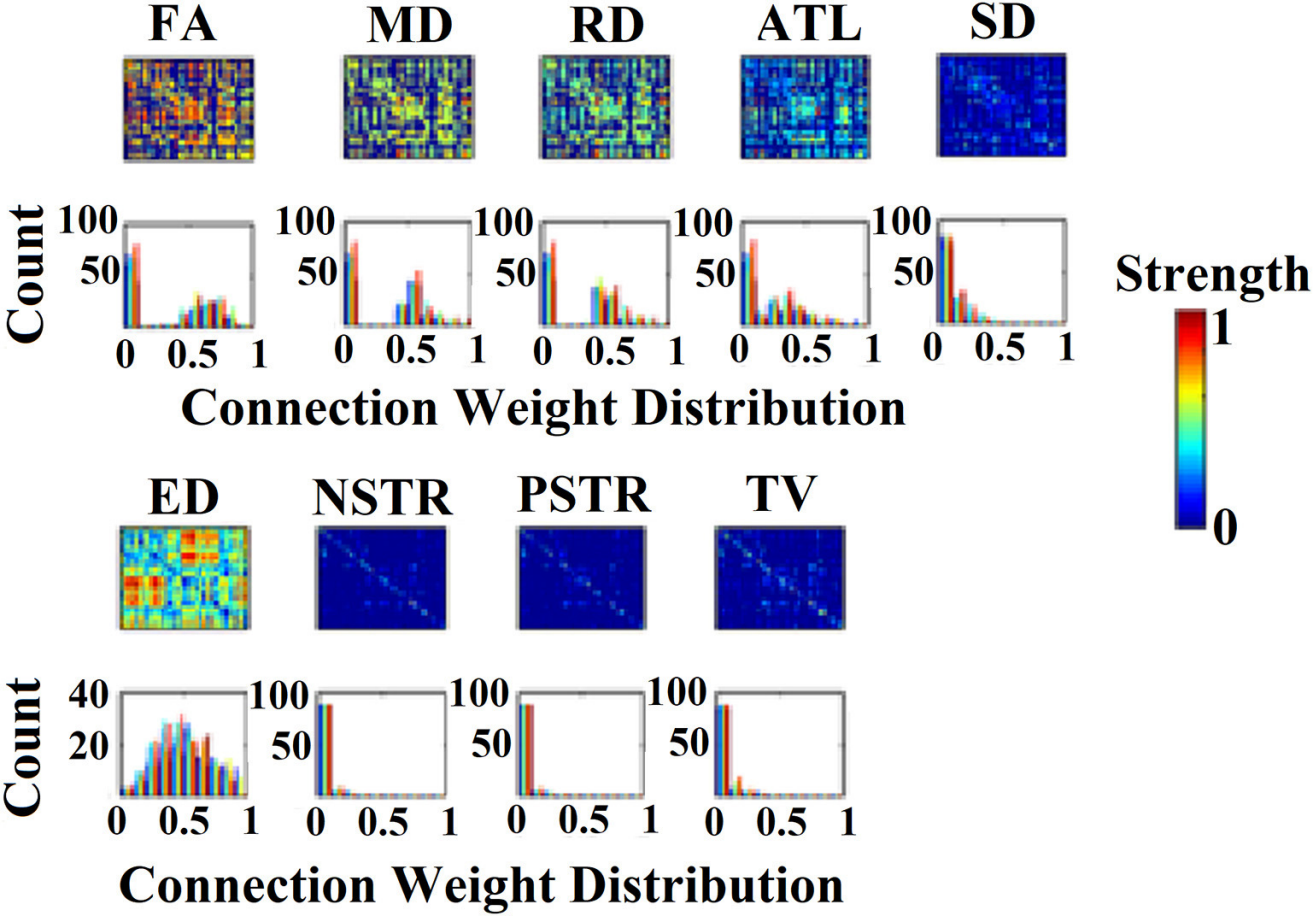
**(Top)** 90 × 90 connectivity matrices of inter-region connections adopted from a subject for the nine network weighting strategies (NWS). **(Bottom)** The corresponding histograms of the connection weights for each of the nine NWS.

Figure 1 illustrates the nine alternative NWS and the corresponding weights from a scan of the first subject.

### Integrating NWS into a single graph

We integrated the different NWS via a linear integration based on the best matching of each pair of NWS-based brain networks in terms of their maximum information flow using the graph diffusion distance metric (gDDM) as described in the next section.

#### Graph diffusion distance metric

We computed the dissimilarity distance between every pair of structural brain networks (SBNs) with a novel gDDM Graph Diffusion Distance (GDD), based on a graph Laplacian exponential kernel (Fouss et al., 2012), served as a distance metric.

The graph Laplacian operator of the SBN was defined as L = D – SBN, where D is a diagonal degree matrix estimated from the SBN. This method entails modeling hypothetical patterns of information flow among sensors based on each observed (static) SBN. The GDD metric reflects the result of the comparison of such patterns between groups. The diffusion process on the person-specific SBN was allowed for a set time t; the quantity that underwent diffusion at each time point is represented by the time-varying vector*u*(*t*)∈ℜ^*N*^. Thus, for a pair of sensors *i* and *j*, the quantity SBN_ij_ *(u*_*i*_*(t) – u*_*j*_*(t))* represents the hypothetical flow of information from *i* to *j* via the edges that connect them (both directly and indirectly). Summing all these hypothetical interactions for each sensor leads to ${u^{\prime }}_{j}(t)=\sum_{i}FCG_{ij}(u_{i}(t)-u_{j}(t))$, which can be written as:

(2) $$u^{i}(t)=-Lu(t)$$

where *L* is the graph Laplacian of SBN. At time *t* = 0, Equation (2) has the analytic solution: *u*(*t*) = exp(−*tL*)*u*^(0)^. Here *exp(-tL)* is a N × N matrix function of t, known as a Laplacian exponential diffusion kernel (Fouss et al., 2012), and *u*^(0)^ = *e*_*j*_, where $e_{j}\in {ℜ}^{N}$is the unit vector with all zeros except in the *j*th component. Running the diffusion process through time t produced the diffusion pattern *exp(–tL)e*_*j*_ which corresponds to the *j*th column of *exp(–tL)*.

Next, a metric of dissimilarity between every possible pair of person-specific diffusion kernelised SBNs (SBN_1_, SBN_2_) in the form of the graph diffusion distance *d*_*gdd*_*(t)* was computed. The higher the value of *d*_*gdd*_*(t)* between the two graphs, the greater the difference in their network topology as well as the corresponding hypothetical information flow. The columns of the Laplacian exponential kernels, *exp(–tL1)* and *exp(-tL2)*, describe distinct diffusion patterns, centered at two corresponding sensors within each SBN. The *d*_*gdd*_*(t)* function is searching for a diffusion time t that maximizes the Frobenius norm of the sum of squared differences between these patterns, summed over all sensors, and is computed as:

(3) $$d_{gdd}(t)={\left\|\exp(-tL_{1})-\exp(-tL_{2})\right\|}_{F}^{2}$$

where ||.||_*F*_ is the Frobenius norm.

Given the spectral decomposition *L* = *V*Λ*V*, the Laplacian exponential can be estimated via:

(4) $$exp(-tL)=Vexp(-t\Lambda )V^{\prime }$$

where for Λ, *exp(–t*Λ*)* is diagonal to the ith entry given by $e^{-t\Lambda_{i,i}}$. We computed d_gdd_(SBN_1_, SBN_2_) by first diagonalizing *L1* and *L2* and then applying Equations (3) and (4) to estimate *d*_*gdd*_*(t)* for each time point t of the diffusion process. In this manner, a single dissimilarity value was computed for each pair of SBNs (Hammond et al., 2013).

#### Linear integration of the different NWS-based SBN into IWSBN

**Figure 2:**
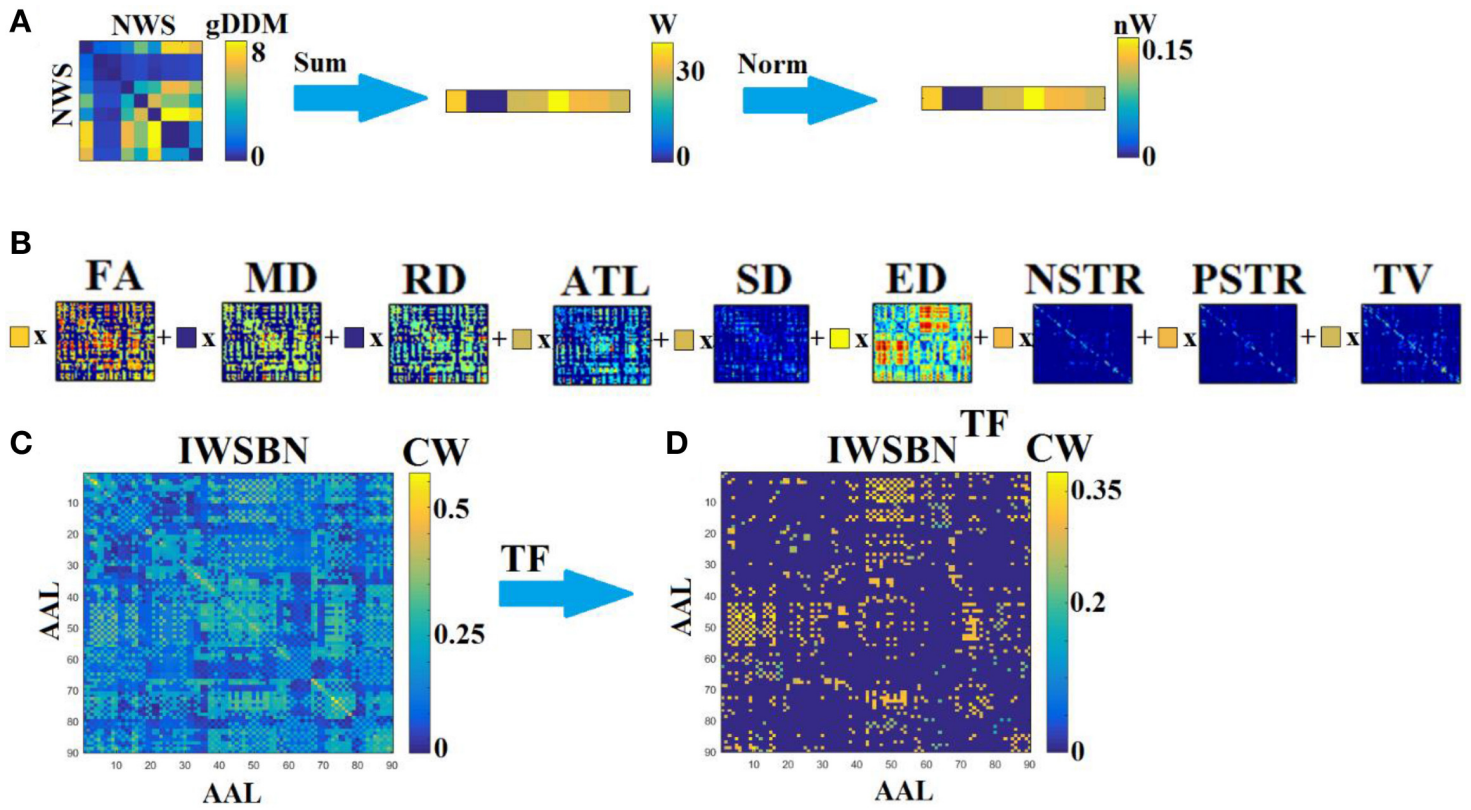
Integrated different network weighting strategies into a single weighted structural brain network (IWSBN). **(A)** From the dissimilarity matrix between every pair of NWS-networks to the related linear weights linked to their interrelationship. We first summed the rows from the dissimilarity matrix *d*^*gDDM*^ and then we normalized these weights *l*_*w*_ such as to have a total sum equal to one. **(B)** Linear integration of the NWS-networks by multiplying (**x**) each NWS-based SBN with the related weight *l*_*w*_ derived from **(A)**. **(C)** The suggested IWSBN derived from **(C)**. **(D)** The topological filtered version of IWSBN called IWSBN^TF^.

**Figure 3:**
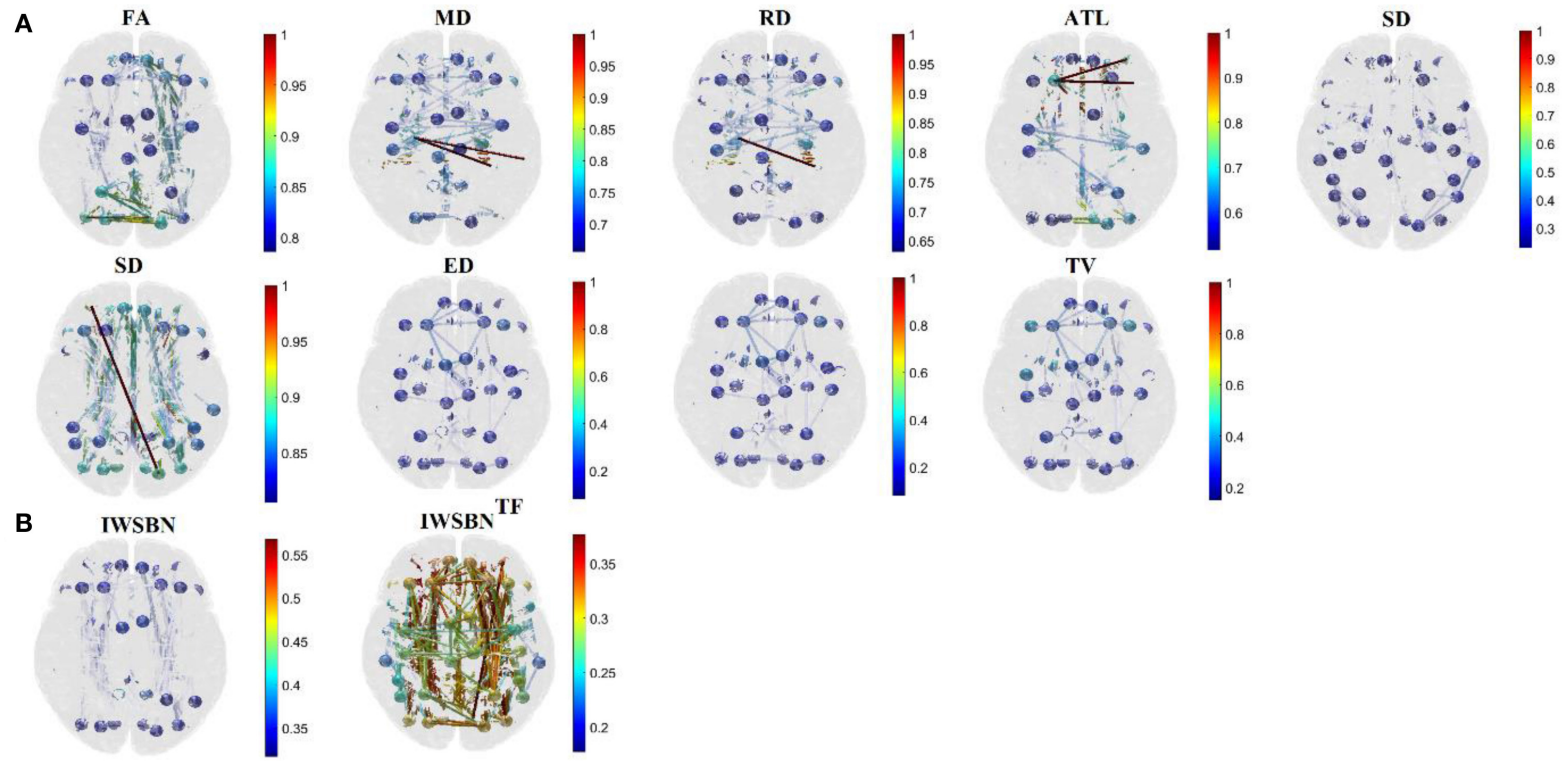
Subject 1—First scan: Topographical layouts of **(A)** nine brain networks derived from the related NWS and the **(B)** IWSBN and its topological filtering version IWSBN^TF^. We plotted the 10% of the strongest connections at each structural brain network to enhance the visualization of the network topology.

Specifically, adopting a gDDM (see previous section Graph Diffusion Distance Metric), we estimated a dissimilarity matrix *d*^*gDDM*^ between every pair of NWS-based brain networks independently for each scan and subject (Figure 2A). Afterward, we estimated the sum of the rows of *d*^*gDDM*^ and then we normalized this derived vector (such as to have a total sum equal to one), to extract weights, *l*_*w*_, for the linear integration of the NWS-based networks into a single graph. Then, we summed across all of these networks weighting each network by *l*_*w*_ (Figure 2B). The result is an IWSBN that is fully-weighted (Figure 2C). Figure 3 illustrates the topologies of the nine NWS from a single subject from their first scan. We plotted the upper decile 10% of the strongest connections according to the related weight.

### Topological filtering of structural brain network

We topologically filtered the IWSBN using a data-driven thresholding scheme that optimizes the information flow over the cost of the surviving/selecting connections. Below, we describe the proposed data-driven topological filtering scheme.

#### Topological filtering based on orthogonal minimal spanning trees (OMST)

In graph theory, a tree is defined as an acyclic connected graph (Estrada, 2011). Acyclic implies that there are no loops (of any length) in the graph. Minimal Spanning Tree (MST) has been shown to be an unbiased, assumption-free method to derive unique functional brain networks (Meier et al., 2015). However, MST is a tree with only *V*-1 links, which for large graphs is too sparse to allow reliable discrimination between two (Antonakakis et al., 2016; Dimitriadis et al., 2017a,b) or more groups (Khazaeea et al., 2017). Two main algorithms have been described to construct the MST of a weighted graph by Kruskal (1956) and Prim (1957). In a recent study, we demonstrated a data-driven topological filtering scheme for brain networks using a large number of EEG and fMRI functional connectivity graphs. Our algorithm samples connections from a fully-weighted graph via OMST (Dimitriadis et al., 2017a,b). The objective criterion was the optimization of the Global Cost Efficiency (GCE) = GE-Cost over each round of the OMST. Cost denotes the ratio of the total weight of the selected edges, over multiple iterations of OMST, divided by the total strength of the original fully-weighted graph. The values of GCE range within the limits of an economical small-world network for healthy control participants (Bassett and Bullmore, 2009). The quality formula is described by the following equation:

(5) $$J_{GCE}^{OMSTs}=GE-Cost$$

**Figure 4:**
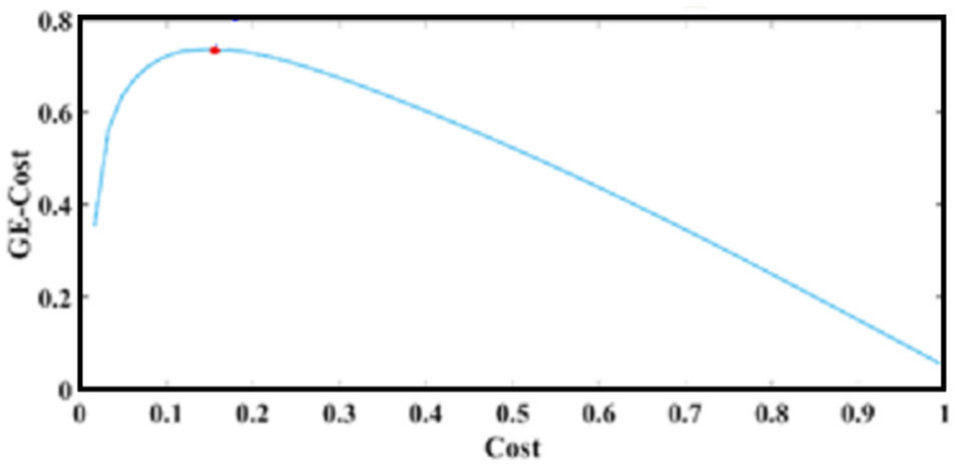
OMST: The optimization of GE-Cost over cost function based on OMSTs from a typical reader. The red circle denotes the peak of the computed curve, while the resulting topologically filtered SBN is shown in Figures 2B,C.

The curve in Figure 4 plots Equation (5) over cost after running exhaustive OMSTs until all observed weights were tested, based on data from a typical reader. The maximum of this (always) positive curve reflects the optimization of the proposed OMST algorithm. In the example of Figure 4, we applied the algorithm in the IWSBN in Figure 2C and the GE-Cost vs. cost function was optimized after four OMSTs leading to a selection of 4^*^89 = 356 connections—a mere 8.9% of the total number of connections that survived the topological filtering approach.

The outcome of this procedure is the IWSBN^TF^ presented in Figures 2D, 3B which is sparser compared to the IWSBN in Figures 2C, 3B. The topological filtering scheme revealed a dense subgraph between frontal areas and calcarine, cuneus, lingual, occipital, and fusiform bilaterally.

### Network measures

For each of the nine weighted SBNs, we estimated six network metrics at the network level and two at the node level. Specifically, for the network level, we estimated global efficiency, local efficiency, characteristic path length, radius, eccentricity and mean weight. For the node level, we estimated global and local efficiency.

### Test-retest statistics

For each metric, an agreement between sessions was computed, via ICC (Shrout and Fleiss, 1979). ICC values were extracted for both network and node level and for every NWS-based brain network, for the IWSBN and also its topological filtering version IWSBN^TF^.

High test-retest reliability is a prerequisite for a connectomic metric to allow for the distinguishing of different individuals (Zuo and Xing, 2014) and also for developing a biomarker of the application of functional connectomics, such as mapping growth charts of human brain function (Dosenbach et al., 2010; Castellanos et al., 2013). Therefore, beyond developing a biomarker, estimations of the test-retest reliability of functional connectomics are valuable for providing a reference regarding how strongly the estimated variables affect the observed results and guiding the significant value of the findings of both normal and abnormal brains (Zuo et al., 2014).

### Classification of structural brain networks

Recognition accuracy was assessed for each individual scan compared to the rest based on a k-nearest neighbor (k-NN) classifier with k = 4 and adopting a leave-one-out cross-validation scheme (LOOCV). Instead of the Euclidean Distance (ED) most commonly used in k-NN classifiers, here we used the proposed gDDM (see section Graph Diffusion Distance Metric). gDDM is a more appropriate metric compared to ED to quantify the distance between two SBNs regarding their distance in terms of information flow based on their topology. The proposed gDDM metric is based on the eigenanalysis of the Laplacian matrices with known attributes in terms of graph theory and diffusion processes (Fouss et al., 2012).

### Discrimination of healthy controls from individuals with psychotic experiences via structural connectome

To demonstrate the effectiveness of the proposed method in a binary classification problem, we analyzed a large dataset consisting of 123 individuals with psychotic experience and 125 age and gender-matched controls. The details of the cohort and the MRI scanning protocol can be found in the original publication (Drakesmith et al., 2015a).

We followed a binary classification procedure with a 10-fold cross-validation, employing as an input, each weighted SBN separately but also the IWSBN and the topologically filtered version (IWSBN^TF^). As a classifier, we used a tensor subspace analysis to reduce the initial high-dimensionality of the original functional connectivity network to a space of condensed descriptive power (Dimitriadis et al., 2015b,c; Antonakakis et al., 2016). The input on TSA is a 3D tensor-matrix of dimensions (subjects × ROIs × ROIs). As a classifier, we used a support vector machine with RBF kernel.

### Exploring the effect of each node to the integrated graph

The proposed IWSBN^TF^ was derived after first linearly combining the nine NWS and after that, topologically filtering the outcome IWSBN. Our second thought was to weight each node independently within each of the nine NWS first and secondly to weight the whole NWS-network with the proposed methodology. To get the linear weights *l*_w_ for each node within each NWS-network, we followed five strategic network lesion schemes, three based node-wise and two cluster-wise.

The first three node-wise strategies were: (1) zeroing half of the connections of each node; (2) diminishing the weights of the connections of each node by 50%; and (3) combining both where half of the connections of each node were zeroed while the weights of the second half were diminished by 50%. The three node-wise attack strategies were followed for one by one node at every NWS and then we estimated the gDDM distance between the original network and the attacked network. Finally, the derived vector with the 90 gDDM values was normalized such as its sum was equal to one. Then, we multiplied each NWS-network node-wise with this vector and afterward with the network-wise approaches based on the present methodology.

The two cluster-wise lesions were: (1) the distinction of the whole set of hubs into rich-club hubs and non-rich-club hubs (van den Heuvel and Sporns, 2011); and (2) the functional clustering of the NWS-network into distinct clusters using the modularity algorithm (Newman, 2006). The two cluster-wise attack strategies were followed for each cluster at every NWS, based on the three node-wise attack strategies targeting either connections within rc-hub subgraphs and/or non-rc-hub subgraph connections and/or the connections between the non-rc and rc-hubs. Then, we estimated the gDDM distance between the original network and the attacked network. Finally, the derived vector with n_cluster_ gDDM values was normalized such as its sum was equal to one.

The whole procedure was added as a first step before the proposed network-wise linear combination of the NWS-network into a single IWSBN All the NWS-networks were pre-filtered with the proposed data-drive thresholding scheme. The node-wise linear weighting step prior to the proposed network-wise was evaluated based on the ICC values of the adopted network metrics both network and node-wise. Additionally, the recognition accuracy of each subject scan over the rest of cohort was compared to the proposed method.

## Results

### Graph embedding of the dissimilarity matrices based on *d^*gDDM*^*

**Figure 5:**
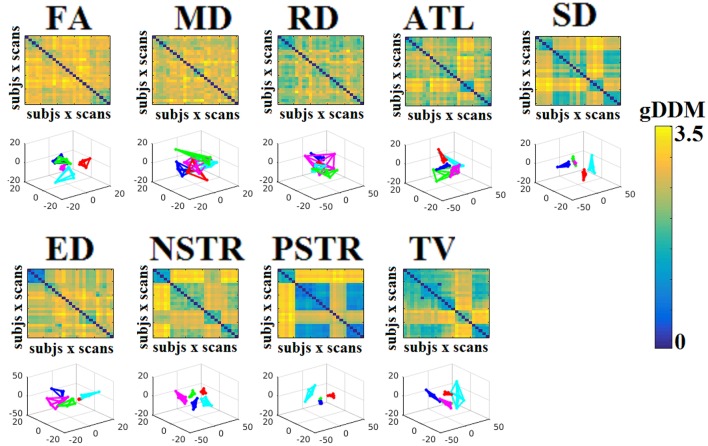
Dissimilarity matrices (DM) based on each NWS across scans and subjects based on the gDDM and graph embedding of DM. First and third rows illustrate the DM between every pair of scans across the cohort based on the gDDM metric for each of the nine NWS, while the second and fourth rows demonstrate the embedded DM via the MDS process in a common 3D space. Each color corresponds to a single subject, while lines with the same color interconnect the NWS derived from repeat scans from the same subject (MDS, multidimensional scaling).

Dissimilarity matrices (DM) based on each NWS across scans and subjects were estimated based on the gDDM. Figure 5 demonstrates the DM for each of the NWS across repeat scans and the related graph embedding based on multidimensional scaling (MDS). The NSTR proved to better discriminate the five subjects compared to the rest of the methods.

(6) $$QCI=\frac{scans\;x\left(scans-1\right)/2}{scans\;x\;scans}\times \frac{\frac{\sum_{su_{1}=1}^{subjects}\sum_{su_{2}=su_{1}+1}^{subjects}\sum_{l=1}^{scans}\sum_{m=1}^{scans}gDDM\left(D\_IWSBN^{TF}\left(su_{1},l\right),D\_IWSBN^{TF}\left(su_{2},m\right)\right)}{subjects^{*}(subjects\;-1)/2}}{\frac{\sum_{su=1}^{subjects}\sum_{l=1}^{scans}\sum_{m=l+1}^{scans}gDDM\left(D\_IWSBN^{TF}\left(su,l\right),D\_IWSBN^{TF}\left(su,m\right)\right)}{subjects}}$$

### Reliability of network level metrics for NWS and ISWBN

**Figure 6:**
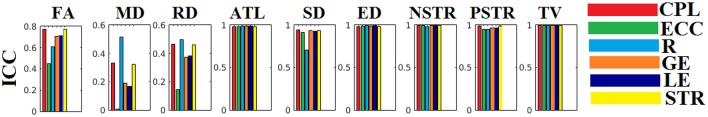
ICC values for basic network metrics on the network level for each of the nine NWS. CPL, Characteristic Path Length; ECC, Eccentricity; R, Radius; GE, Global Efficiency; LE, Local Efficiency; STR, mean strength.

**Figure 7:**
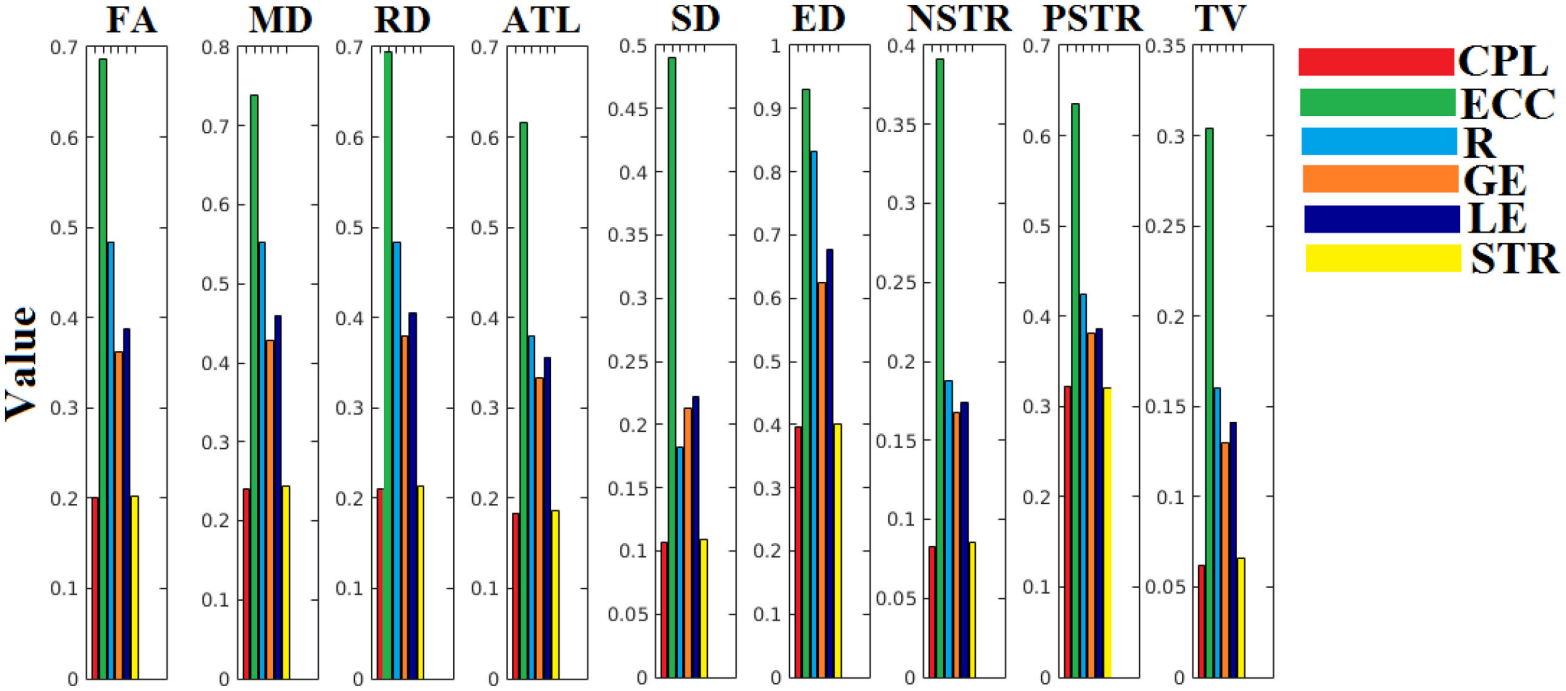
Group-averaged values of the adopted network metrics at each NWS. CPL, Characteristic Path Length; ECC, Eccentricity; R, Radius; GE, Global Efficiency; LE, Local Efficiency; STR, mean strength.

**Figure 8:**
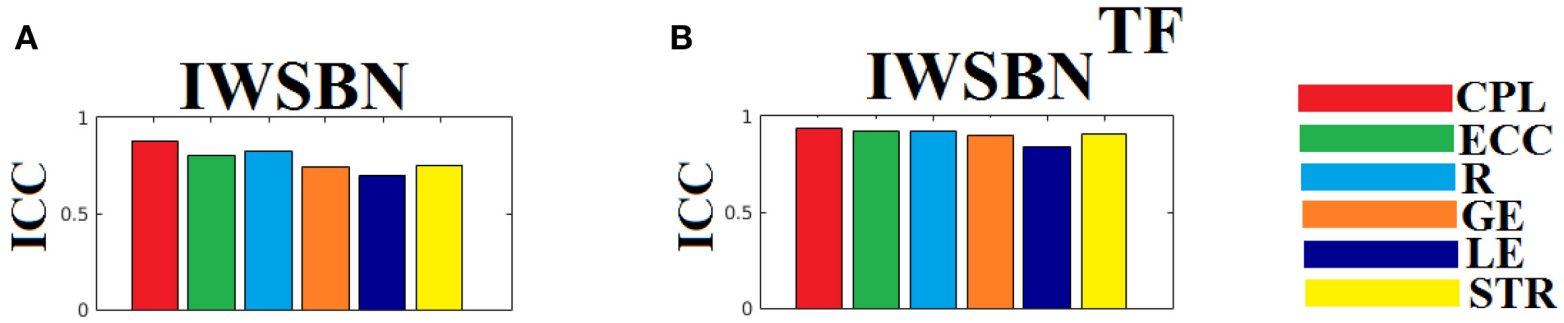
ICC values for basic network metrics on the network level for both **(A)** IWSBN and **(B)** IWSBN^TF^. CPL, Characteristic Path Length; ECC, Eccentricity; R, Radius; GE, Global Efficiency; LE, Local Efficiency; STR, mean strength.

**Figure 9:**
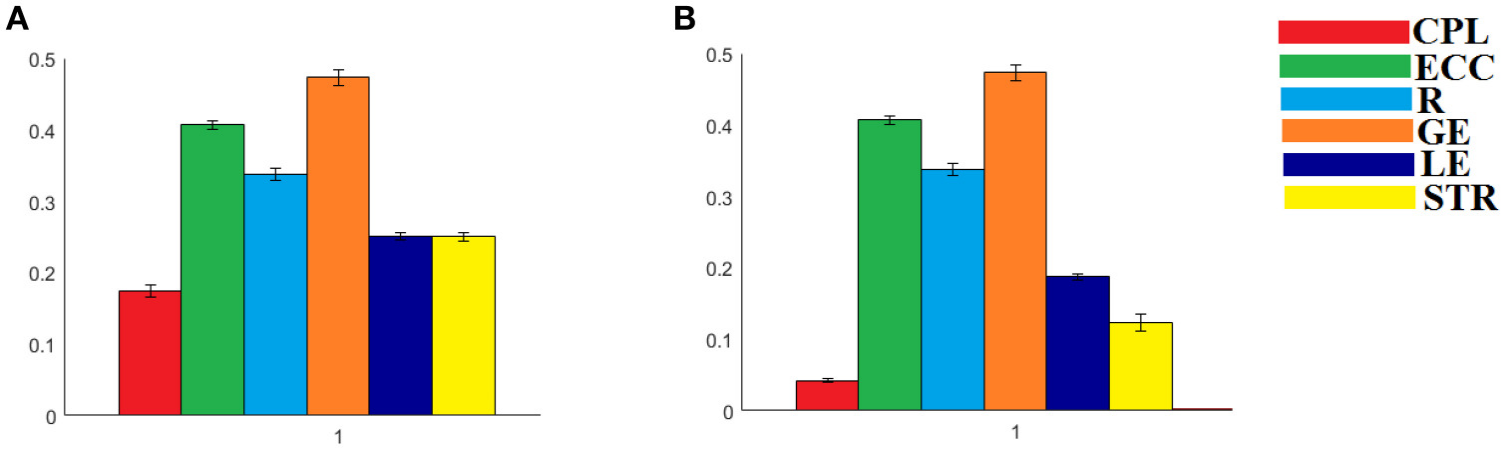
Group-averaged values of the adopted network metrics for both **(A)** IWSBN and **(B)** IWSBN^TF^. CPL, Characteristic Path Length; ECC, Eccentricity; R, Radius; GE, Global Efficiency; LE, Local Efficiency; STR, mean strength.

The ICC scores were excellent—ranging from 0.75 to 1—for six out of nine network metrics for the entire set of network metrics. These NWS include the ATL, BIN, SD, ED, NSTR, PSTR, and TV (Figure 6). The related group-averaged values of the network metrics for each NWS are shown in Figure 7. The proposed IWSBN yielded good ICC values but these were lower than those obtained for each of the six NWS (Figure 8A). Significantly, we observed an improvement of the ICC on the IWSBN^TF^ which reached the level of the six best NWS in terms of ICC scoring (Figure 8B). Figure 9 demonstrates the group-averaged values of network metrics on the network level.

### Reliability of network metrics on a node level

**Figure 10:**
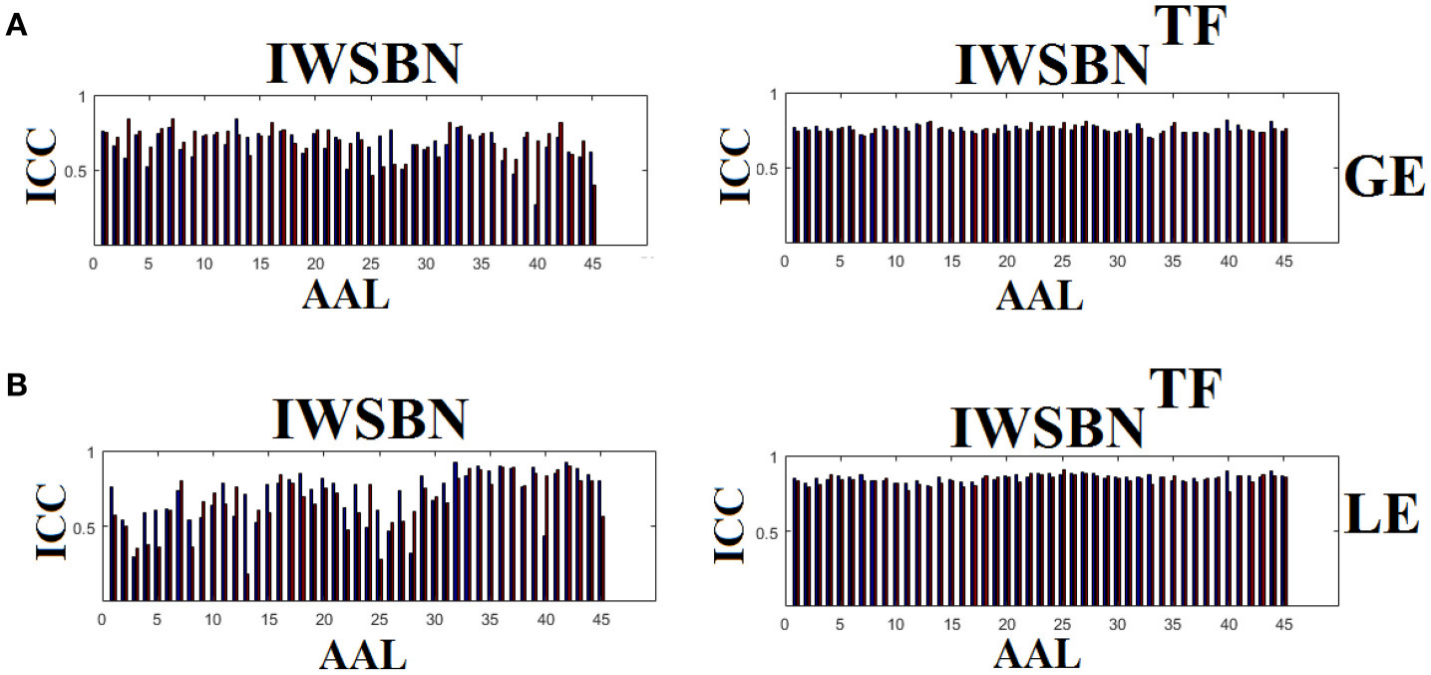
ICC values for global and local efficiency on the node level for both IWSBN and IWSBN^TF^. **(A)** ICC node-wise score for global efficiency on **IWSBN and IWSBN**^TF^. **(B)** ICC node-wise score for local efficiency on **IWSBN and IWSBN**^TF^. Blue/red bars refer to Automated Anatomical Labeling (AAL) ROIs for left/right hemisphere correspondingly.

**Figure 11:**
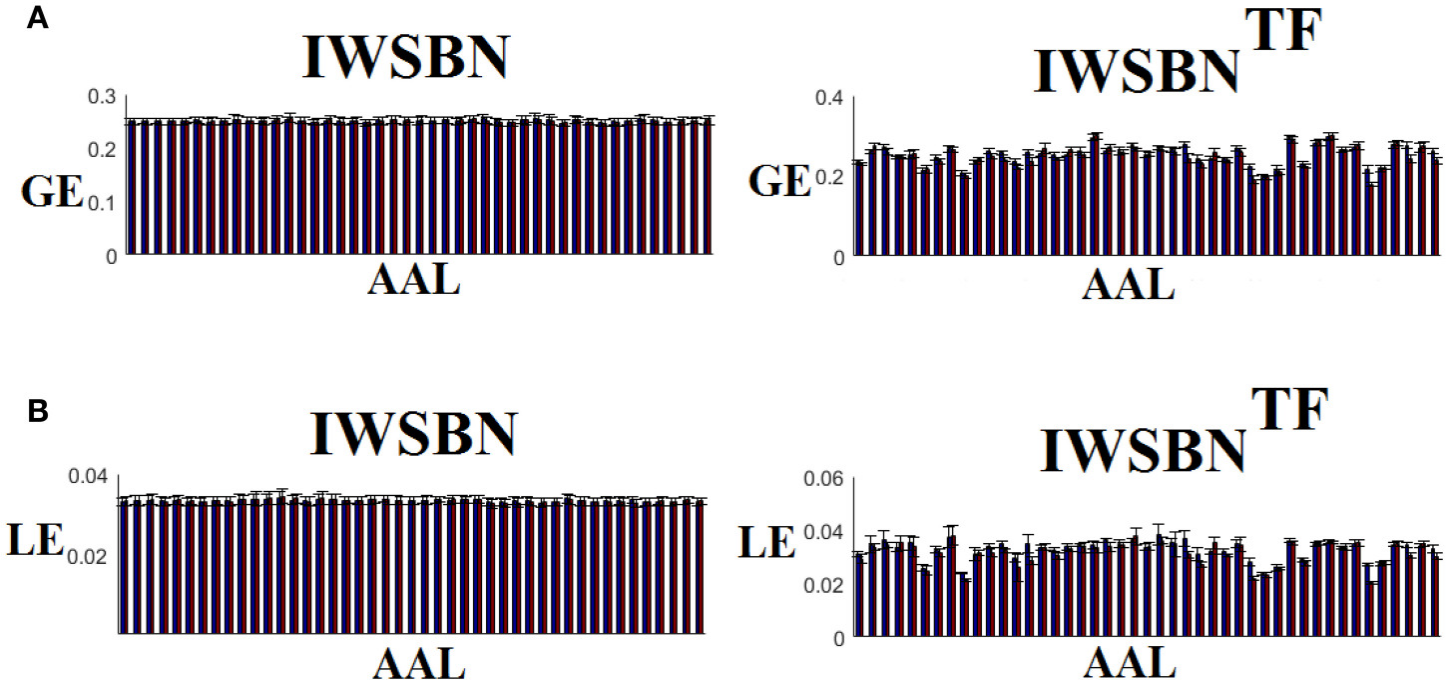
Mean/std values for global and local efficiency on the node level for both IWSBN and IWSBNTF. **(A)** Mean/Std node-wise GE for global efficiency on **IWSBN and IWSBN**^TF^. **(B)** Mean/Std node-wise LE for local efficiency on **IWSBN and IWSBN**^TF^. Blue/red bars refer to AAL ROIs for left/right hemisphere correspondingly.

The analysis of ICCs on global and local efficiency node-wise on the nine NWS and in both IWSBN and IWSBN^TF^ revealed important trends. Firstly, the ICC values for each of the NWS failed to reach a fair value (ICC < 0.1). Secondly, the ICC values derived from the IWSBN showed a large variability but reached on average ICC = 0.68 ± 0.10 for global efficiency and ICC = 0.68 ± 0.17 for local efficiency (Figure 10A). Third, the ICC values for both network metrics were improved in IWSBN^TF^, reaching a mean ICC = 0.75 ± 0.02 for global efficiency and a mean ICC = 0.84 ± 0.02 for local efficiency (Figure 10B). Applying a Wilcoxon Rank Sum Test between the two distributions for each network metrics, we observed significant improvement of ICC values for IWSBN^TF^ (global efficiency: *p* = 0.0035 × 10^−7^, local efficiency: *p* = 0.0067 × 10^−10^). Figure 11 demonstrates the group-averaged values of network metrics on the node level.

### Recognition accuracy of structural brain networks

**Figure 12:**
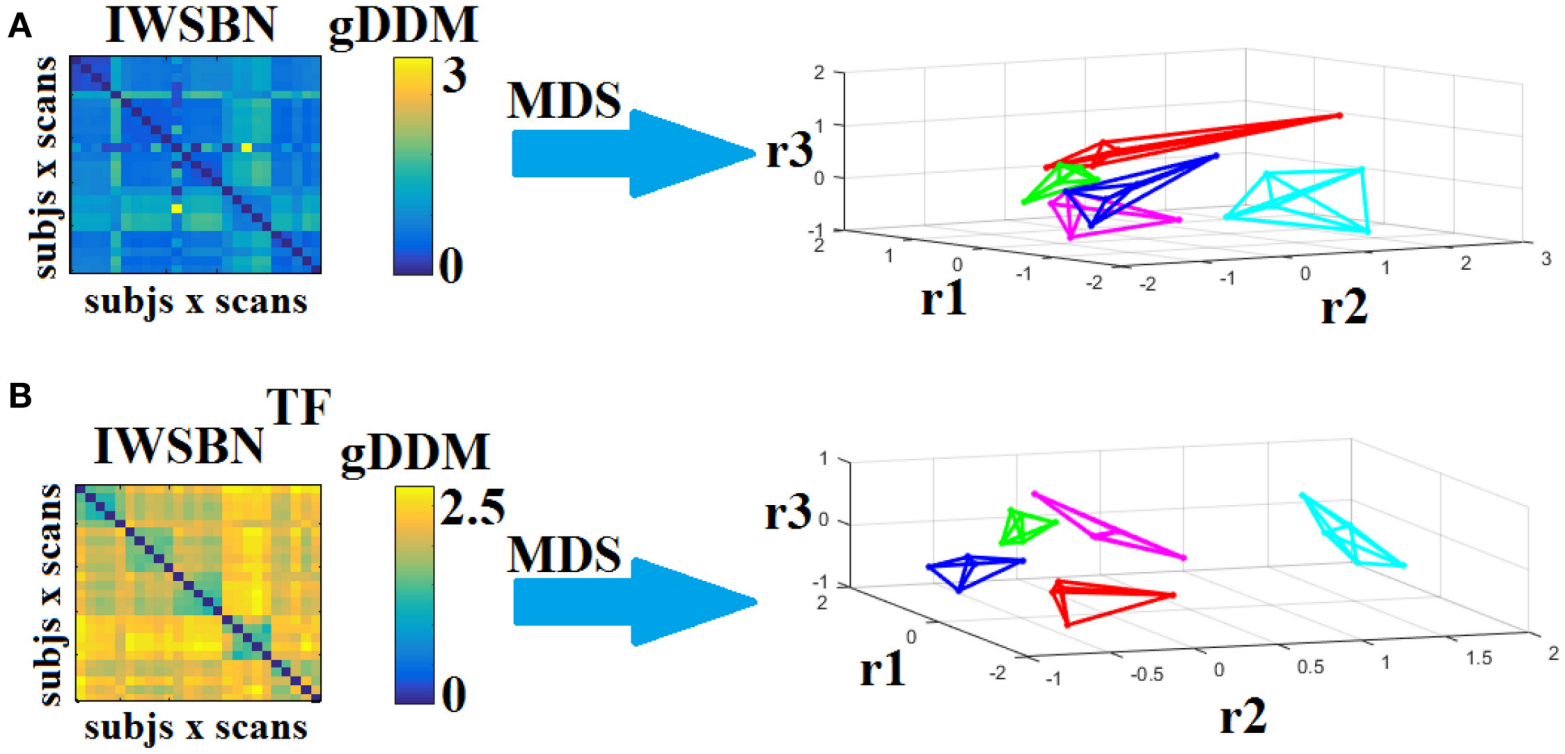
Dissimilarity matrices (DM) based on both IWSBN and IWSBN^TF^ across scans and subjects using gDDM and graph embedding of DM. **(A)** The first row illustrates the DM based on the IWSBN and its embedding in a 3D space, while the **(B)** second row demonstrates the DM based on the IWSBN^TF^ and its embedding in a 3D space. Each color corresponds to a single subject, while lines with the same color interconnect the NWS derived from repeat scans (MDS, multidimensional scaling).

Dissimilarity matrices (DM) based on both IWSBN and IWSBN^TF^ across scans and subjects were estimated based on the gDDM. Figure 12 demonstrates the DM for both IWSBN and IWSBN^TF^ across repeat scans and the related graph embedding based on multidimensional scaling (MDS). The proposed topological filtering scheme improved the discrimination of the five subjects compared to the original IWSBN.

Applying a k-NN classifier with *k* = 4 and gDDM as the appropriate distance metric under a LOOCV scheme, we succeeded to accurately classify each scan to the right person based on IWSBN^TF^. Similar results were also obtained with NSTR (Figure 5).

For a better estimation of the discrimination of proposed IWSBN^TF^ with NSTR, we defined the following quality of clustering index (QCI) (Dimitriadis et al., 2012):

The QCI quantifies the average similarity of the IWSBN^TF^ across scans within each subject (cluster) expressed in the denominator of Equation (6) and the average dissimilarity between every pair of subjects (clusters) across their scans expressed in the numerator. Both similarity and dissimilarity were estimated based on the gDDM. The higher the dissimilarity between the clusters (numerator) and/or the lower the dissimilarity within the clusters (denominator), the higher the QCI. The numerator is averaged across all possible combinations of subjects (clusters) while the denominator across subjects (clusters). The first term was used to equalize the effect of between-subject (clusters) comparisons vs. within-subject comparisons (clusters). This inversed coefficient guarantees that in the case of all the weights in the DM being equal then it will take a value of one. Therefore, the higher the value of the QCI above one, the higher the separability between the network topologies of the subjects.

We first tabulated all the IWSBN^TF^ across scans and subjects into a 4D graph with dimensions equal to [subjects × scans × Rois × Rois] called D_ IWSBN^TF^. Afterward, we estimated the QCI for each NWS and for both IWSBN and IWSBN^TF^.

The QCI was 1.45 for IWSBN and 6.45 for IWSBN^TF^ while for NSTR the QCI was 4.57. This result can be interpreted as a higher separation of network topologies with our approach compared to the best of NWS.

**Figure 13:**
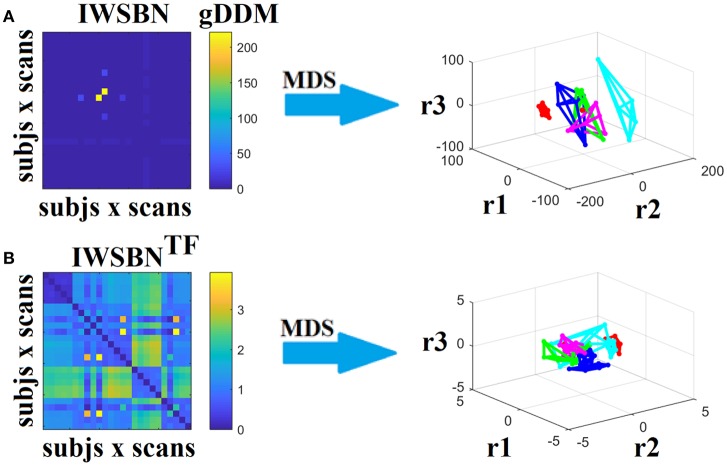
Dissimilarity matrices (DM) based on both IWSBN and IWSBN^TF^ across scans and subjects using gDDM and graph embedding of DM (as in Figure 12). Both **(A)** IWSBN and **(B)** IWSBN^TF^ were constructed based on FA, MD, and RD weighted structural brain networks.

**Figure 14:**
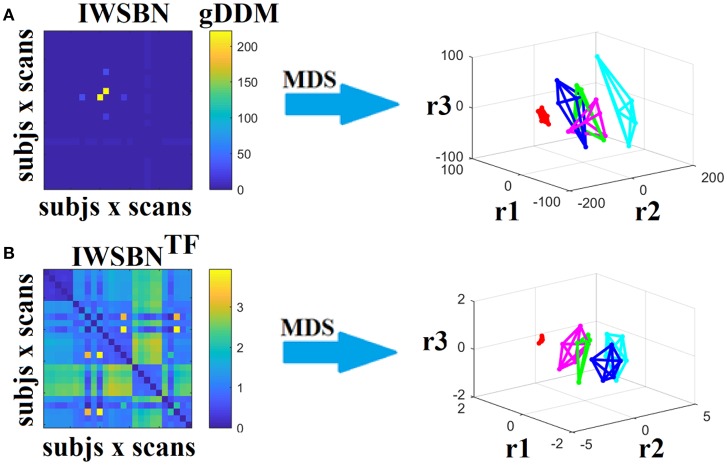
Dissimilarity matrices (DM) based on both IWSBN and IWSBN^TF^ across scans and subjects using gDDM and graph embedding of DM (as in Figure 12). Both **(A)** IWSBN and **(B)** IWSBN^TF^ were constructed based on ATL, SD, and ED weighted structural brain networks.

**Figure 15:**
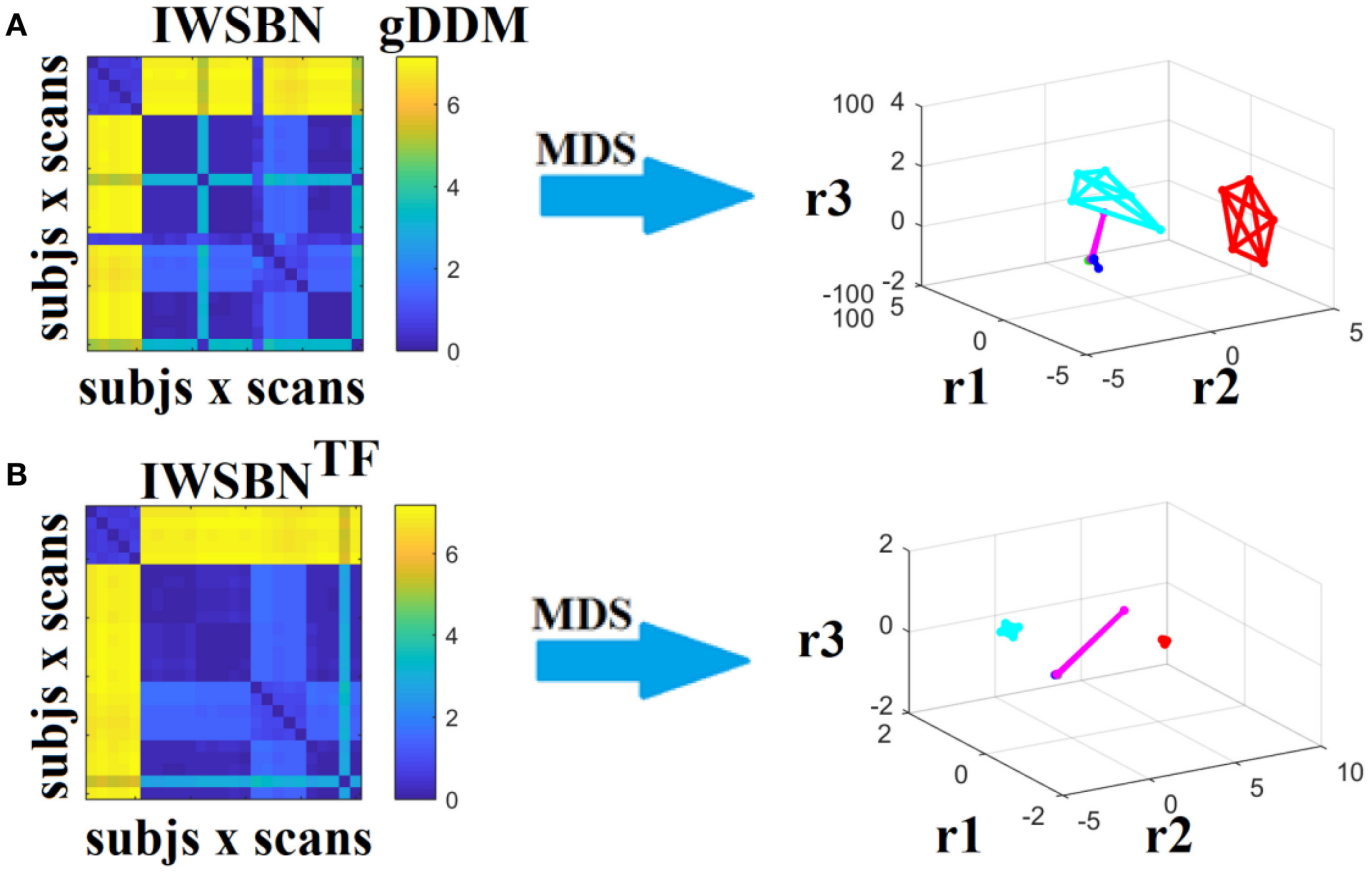
Dissimilarity matrices (DM) based on both IWSBN and IWSBN^TF^ across scans and subjects using gDDM and graph embedding of DM (as in Figure 12). Both **(A)** IWSBN and **(B)** IWSBN^TF^ were constructed based on NSTR, PSTR, and TV-weighted structural brain networks.

To further enhance the integration of the nine alternative WNS for the construction of an integrated SBN, we repeated the whole procedure splitting the nine weighted versions of SBN into three triads (the first three, the second three and the last three). Figures 13–15 illustrate the DM and their embedding into a 3D-space. The highest separability between the network topologies of the subjects have been demonstrated for ATL, SD, and ED, while the worst for NSTR, PSTR, and TV, where three subjects overlapped on the same embedding space (Figure 15B). The QCI score was lower compared to the original approach where we combined the nine weighted SBN (Figure 12).

### Structural connectomic classification of healthy controls (HC) from individuals with psychotic experiences (PE)

Our classification results demonstrated a higher classification accuracy (65.3%) between the two groups for the proposed IWSBN^TF^. The classification performance of the nine weighted strategies was lower than by chance (<50%; see Table 1). Additionally, the data-driven topological filtering via the OMST algorithm (Dimitriadis et al., 2017a,b) further improved the classification accuracy (from 57.23 to 65.34%; see Table 1).

**Table 1:** Accuracy, sensitivity, and specificity of the nine weighted strategies, the IWSBN and the IWSBN^TF^ following a binary classification of HC vs. individuals with PE via a 10-fold cross-validation strategy.

**LOOCV**	**Accuracy**	**Sensitivity**	**Specificity**
FA	43.44 ± 5.43	42.12 ± 4.99	42.19 ± 5.08
MD	42.81 ± 4.82	42.72 ± 5.12	43.06 ± 4.91
RD	44.17 ± 5.67	43.65 ± 4.92	43.57 ± 5.24
ATL	45.78 ± 5.61	44.73 ± 5.32	44.82 ± 5.39
SD	45.81 ± 6.71	44.89 ± 5.21	44.71 ± 5.96
ED	46.07 ± 5.92	45.39 ± 5.66	45.87 ± 5.23
NSTR	45.91 ± 5.11	45.44 ± 5.43	45.21 ± 4.89
PSTR	46.17 ± 5.42	46.79 ± 6.13	45.94 ± 5.69
TV	46.38 ± 5.76	46.45 ± 6.32	45.82 ± 5.73
IWSBN	57.23 ± 6.89	56.79 ± 6.20	55.89 ± 5.81
IWSBN^TF^	**65.34** ± **6.71**	**64.36** ± **5.87**	**65.04** ± **6.05**

*We underlined with bold font the best classification accuracy succeeded with the proposed IWSBN^TF^*.

## Discussion

We present, for the first time, the reliability of basic network metrics at both whole-network and node level for nine different NWS. We recruited five subjects who were scanned five times at weekly intervals. The range of age was (mean 37.1 ± 4.9 years of age, five males) to minimize the effect of the age on inter-subject variability. Additionally, for the first time, we propose a completely data-driven algorithm for the linear interpolation of the different NWS-based SBNs into a single IWSBN. The whole approach is based on a diffusion distance metric that quantifies the maximum distance between two network topologies in terms of their information flow. Complementarily, we propose a completely data-driven topological filtering scheme for extracting the backbone of a SBN on an individual level (scan-based) without attempting to find any consistency among control subjects of a specific age (Roberts et al., 2016). To reveal any gender, age or even individualized differences in terms of dMRI-based SBNs, we should adopt data-driven techniques applied to individual SBNs without any a priori knowledge of the label of a subject's scan (age, gender, HC). Any adopted group or scan consistency as a constraint to the main methodology will diminish individual differences and across scan variability, respectively. Our results can be summarized into the following key points:

- ICC values of network metrics derived from network levels were high for six out of nine NWS
- ICC values of network metrics derived node-wise were unreliable for all nine NWS
- ICC values for all the network metrics on the network level for IWSBN^TF^ were excellent and on the same level as the best NWS
- We observed high ICCs of network metrics node-wise for both IWSBN and IWSBN^TF^ compared to each NWS with higher values succeeding based on IWSBN^TF^
- We succeeded in achieving a higher discrimination of each subject compared to the rest of the cohort based on the IWSBN^TF^ derived from each scan compared to IWSBN and the best NWS which was the NSTR
- The construction of subject-specific IWSBN^TF^ for two large populations (HC and individuals with PE) further improved the classification performance compared to each of the nine weighted versions of their structural connectome.

Previous studies explored different aspects of network reliability using repeat dMRI scans of healthy human volunteers. Hagmann et al. (2008) assessed structural networks obtained from diffusion spectrum imaging (DSI), while Vaessen et al. (2010) assessed reproducibility over different sets of diffusion gradient directions using DT-MRI. Bassett et al. (2011) compared reliability in both DT-MRI and DSI, and Cammoun et al. (2012) investigated the effect of network resolution using DSI. Finally, Cheng et al. (2012) assessed test-retest reliability using DT-MRI, with a focus on the differences between binary and weighted networks. A recent study explored the reliability of network metrics on a network and node-wise level using dMRI and two alternative tractography algorithms, two alternative seeding strategies, a white matter way point constraint and three alternative network weightings (Buchanan et al., 2014). Their best performing configuration, the global within-subject differences, showed ICCs between 0.62 and 0.76, while the mean nodal within-subject differences demonstrated ICCs between 0.46 and 0.62. In the present study, we revealed higher ICC values, both node and network-wise, based on the IWSBN^TF^. Furthermore, applying our network analysis on IWSBN^TF^, we observed higher between-subject differences compared to within-subject variation (see Figure 9B).

Buchanan et al. (2014), concluded that regional reliability of dMRI networks is low suggesting that connections between specific pairs of nodes are unreliable across sessions. Here, we showed the same issue for each of the nine NWS leading to very small ICCs (<0.1). Applying the proposed topological filtering scheme to each of the NWS, we failed to further improve the nodal ICC, which can be interpreted as technical issues derived from tractography (data not shown). Errors in tractography in estimating axonal tracts may reflect both the segmentation of each ROI affecting the streamline construction. Tractography is strongly affected by measurement noise resulting in both false negative and positive connections (Zalesky and Fornito, 2009). Yo et al. (2009) compared different tractography algorithms focusing on the uncertainty of fiber directions in a noisy environment which could be a factor for poor ICC values for node-wise estimated network metrics.

Common poor ICC values for node-wise network metrics for each of the nine NWS and simultaneously excellent ICC values for network-wise network metrics for six out of nine NWS could be interpreted as a common error of the tractography for the former and as a denoising procedure from the latter after integrating across all nodes. It seems that the proposed dual-step scheme for combining NWS into a single IWSBN^TF^diminished any bias of probabilistic tractography and led to a reliable nodal ICC which was higher than that demonstrated in previous work (Buchanan et al., 2014). Both steps proved crucial to simultaneously elevating the ICC values of network metrics network-wise to excellent levels—comparable to the best NWS—and the ICC node-wise values linked to network metrics to fair to good levels (see Figure 8).

It is important to mention here that Buchanan et al. (2014) preferred not to threshold the derived weighted SBNs in order to avoid biasing their results. We completely agree with this approach since, until now, none of the non-data-driven thresholding schemes can work without any bias selection of any criterion. With the present study, we proposed a solution for uncovering the backbone of a SBN by increasing the information flow within the network constrained by the overall cost of the selected connections.

The majority of studies focusing on SBNs have worked on region-to-region connectivity using an anatomical map while they ignored the rich information in the local white matter architecture (Yeh et al., 2016). In this study, the authors analyzed local connectomes, termed connectometry, which tracks the local connectivity patterns along the fiber pathways to further extract the subcomponents of the pathways that are associated with the parameters of study. They demonstrated that connectometry complements global brain networks while they are more sensitive and less affected by fiber tracking issues.

The proposed IWSBN^TF^ was derived after first linearly combining the nine NWS and then topologically filtering the derived IWSBN. Our second thought was to independently weight each node within each of the nine NWS first and secondly to weight the whole NWS-network with the proposed methodology. The whole procedure was added as a first preliminary step before the proposed network-wise linear combination of the NWS-network into a single IWSBN. The topological filtering of each NWS-network prior to the node and cluster-wise attacking strategies did not improve the ICC values of network metrics, neither network or node-wise. Additionally, the recognition accuracy was worse compared to the proposed method. One possible interpretation of these results could be that specific connections cause a major effect on the reliability of the whole-network topology. Future strategic artificial lesion approaches on a connection level could reveal where (anatomically) and when (protocols, scanners, other factors) a tractography algorithm produces errors. This methodology could be useful to improve the algorithm between specific tracts.

Future study will shed light on how the proposed dual-step methodology can affect the reliability of connectomic biomarkers in conditions such as Alzheimer's Disease, schizophrenia with a genetic background and dyslexia. Here, we demonstrated the effectiveness of the proposed methodology in discriminating HC from individuals with PE. Additionally, we will compare IWSBN^TF^ between different dMRI protocols and also between different scanners with the same or different field strengths (3T and 7T). Finally, large publicly-available dMRI cohorts can be analyzed with this method in order to reveal developmental trends. For all these research questions—looking at individual differences, longitudinal trajectories and case-control difference—a high degree of reliability of the underlying metrics is crucial, and thus our approach could be widely adopted. This data-driven topological filtering algorithm can be a baseline across different studies and big datasets e.g., the Human Connectome Project, UK BIOBANK in order to share metadata in a common feature space across institutes, research centers, universities and research groups.

### Motivations derived from the current study

Analysis of reliability is challenging for neuroimaging as the main results presented in a study depend on both the adopted network metrics and the metrics used to characterize the weight of a connection. Additionally, many neuroimaging studies based on SBNs attempt to shed light on developmental differences, differences between clinical populations and also between a control group and a disease group. A basic reason why all these proposed connectomic biomarkers are not used in daily practice in hospitals is their reliability (Dimitriadis et al., 2015a). A second reason is that there are many studies on the same topic (e.g., brain disease) based on small datasets that adopted different NWS and arbitrary topological filtering schemes. Meaningful aggregation of these, even in the case that their metadata are free available, is impossible. A third reason is that until now, it is not standard practice to assess the reliability of network metrics derived from SBNs across repeat scans on the same population but with different dMRI protocols and scanners (3T vs. 3T or 3T vs. 7T). This is an issue that we would like to investigate in future studies with the proposed scheme.

A basic issue with the ICC is that it needs large samples in order to estimate scores to acceptable precision. A study estimated that for two repeated measures, in order get an acceptable ICC score of 0.8 with a 95% confidence interval of 0.2 width, then at least 52 subjects are needed (see Table 3 in Shoukri et al., 2004). In a similar vein, for an ICC score of 0.6 with 95% confidence intervals of 0.2 width, repeated measures from 158 subjects would be required. Clearly for most MRI-based studies, this scenario of repeated scans for hundreds of scans is unrealistic. However, our analysis provided a methodology of how to combine different NWSs into a single integrated graph based on a gDDM that counts the network topology as a whole and quantifies the distance between two SBNs in terms of their information flow. The results of the proposed methodology presented here, even in a small dataset, are of paramount importance since they are completely data-driven in any preprocessing step. Simultaneously, they provided novel directions of how to untangle hidden information within dMRI-based brain networks by working on an individualized manner and without any averaging approach (group or scan-wise). Additionally, we proposed a data-driven thresholding scheme applied to the IWSBN that improved the ICCs of basic network metrics at both node and network levels compared to the original IWSBN and each of the adopted NWS. Our data-driven method can be seen as a methodology for improving network reliability on SBNs (Zalesky et al., 2010; Drakesmith et al., 2015b). Complementarily, our approach provided excellent discrimination of the network patterns of the five subjects based on the recognition of each scan to the targeted subject. Finally, our method better separated the five groups of scans based on the topological filtering version of IWSBN compared to the best NWS.

### Limitations of the study

It is important to mention here the limitations of the current study due to the small dataset for exploring the test-retest reliability statistics. In the era of open science resource where multisite worldwide neuroimaging labs share neuroimaging datasets, it is important to demonstrate novel techniques that improve the reliability of connectomics in common neuroimaging data (Zuo et al., 2014). A recent Consortium for Reliability and Reproducibility (CoRR) is working to address this gap and establish test-retest reliability as a minimum standard for methods development in functional connectomics (Zuo and Xing, 2014; Zuo et al., 2014) and morphological measurements (MacLaren et al., 2014). Reliability is important to build reliable connectomic biomarkers across multi-site (Nielsen et al., 2013; Abraham et al., 2017) and also longitudinal trajectories of structural and functional brain networks across the life-span (Zuo et al., 2017).

Our future goal is to test the proposed methodology in a larger sample for validating the test-retest reliability of our scheme and also on multi-site diffusion-based structural brain networks for building reliable connectomic biomarkers.

## Conclusion

Reliability analysis of both node and network-wise network metrics in IWSBN and its topological filtering version revealed: (1) similar ICC values for all the network metrics on the network level for IWSBN^TF^ compared to the best NWS; (2) higher ICC of network metrics node-wise for both IWSBN and IWSBN^TF^ compared to each NWS with higher values succeeding based on IWSBN^TF^; and (3) higher discrimination of each subject compared to the rest of the cohort based on the IWSBN^TF^ derived from each scan compared to IWSBN and the best NWS which was the NSTR. We thus provided a new approach to identifying highly reliable and discriminative network metrics that can be the basis for studies of interindividual differences, longitudinal trajectories, and pathological changes in structural brain connectivity.

## Ethics statement

This study was approved by the ethical committee in the Cardiff University.

## Author contributions

SD: conception of the research, methods and design, and drafting the manuscript; SD, MD, GP, SB: data analysis; MD, GP, SB, DL, DJ: critical revision of the manuscript; Every author read and approved the final version of the manuscript.

### Conflict of interest statement

The authors declare that the research was conducted in the absence of any commercial or financial relationships that could be construed as a potential conflict of interest.
